# Systematic Evaluation of Gastric Tumor Cell Index and Two-Drug Combination Therapy via 3-Dimensional High-Throughput Drug Screening

**DOI:** 10.3389/fonc.2019.01327

**Published:** 2019-11-29

**Authors:** Sung Hee Lim, Jason K. Sa, Dong Woo Lee, Jusun Kim, Seung Tae Kim, Se Hoon Park, Bosung Ku, Joon Oh Park, Young Suk Park, Hoyeong Lim, Won Ki Kang, Do-Hyun Nam, Jeeyun Lee

**Affiliations:** ^1^Division of Hematology-Oncology, Department of Medicine, Samsung Medical Center, Sungkyunkwan University School of Medicine, Seoul, South Korea; ^2^Division of Hematology-Oncology, Department of Medicine, Soon Chun Hyang University Hospital Bucheon, Bucheon-si, South Korea; ^3^Department of Biomedical Sciences, Korea University College of Medicine, Seoul, South Korea; ^4^Department of Biomedical Engineering, Konyang University, Daejeon, South Korea; ^5^Institute for Refractory Cancer Research, Samsung Medical Center, Seoul, South Korea; ^6^Department of Neurosurgery, Samsung Medical Center, Sungkyunkwan University School of Medicine, Seoul, South Korea

**Keywords:** tumor heterogeneity, tumor purity, patient-derived tumor cell, high-throughput drug screening, two-drug combination

## Abstract

Tumor heterogeneity greatly limits personalized treatment of cancer. Patient-derived tumor cell (PDC) models precisely recapitulate the molecular properties and biology of the disease, making them effective preclinical tools for assessing anti-cancer drug activities. Accurate estimation of tumor purity is essential for performing high-throughput drug screening (HTS). In the present study, we measured and predicted the tumor population index in PDC models for two-drug combinational strategies using HTS system. Gastric cancer cell-lines and PDCs were subjected to multi-color immunofluorescence analysis against EpCAM and vimentin to evaluate the tumor cell index based on EpCAM expression levels. We generated a tumor purity prediction model using five different gastric cancer cell-lines (AGS, KATO-III, MKN-45, NCI-N87, SNU-216) with fluorescence intensity-based techniques. Afterwards, stage IV gastric cancer PDC models were evaluated using a micropillar/microwell chip-based HTS system. HER2/CCNE1-amplified PDCs were considerably resistant to an HER2 inhibitor, while combinational treatment consisting of an HER2 inhibitor with anti-WEE1 compound substantially suppressed tumor cellular growth. Moreover, PDCs with *BRCA1/2* mutations were synergistically sensitive to HER2 and PARP inhibition therapy. Finally, somatic mutations in *TP53* and *CDKN2A* with *MYC* amplification rendered PDCs susceptible to the drug combination of WEE1 and HER2. Collectively, our systematic method of high-throughput drug sensitivity screening is an integral pre-clinical platform for evaluating potential two-drug combinational approaches for personalized treatment of cancer.

## Introduction

Gastric cancer is the third leading cause of cancer-related mortality. The current standard-of-care for patients with gastric cancer provides only palliative treatment despite the availability of curative surgery (1). Although previous studies performed comprehensive molecular characterization of gastric adenocarcinomas based on four specific molecular subtypes (2, 3), the clinical application potential of molecular targeted therapy for personalized treatment remains unclear. Based on the clinical success of trastuzumab in patients with HER2-overexpressing advanced gastric cancer (4), numerous phase III clinical trials, concomitant with other targeted agents, have been initiated, but have shown disappointing outcomes (5–11). An essential contributing factor to such termination is the extensive molecular and transcriptional heterogeneity of gastric cancer.

Conventional pre-clinical tools for evaluating pharmacological drug responses primarily rely on two-dimensional cultured cancer cell-lines or patient-derived xenograft (PDX) models. While both models have been used widely and extensively for translational cancer research, several challenges limit their direct clinical utility. First, traditional cancer cell-lines may not recapitulate the unique genetic background of each patient. Second, while PDX systems retain the genomic characteristics of the parental tumors *in situ*, generation of PDX models is often resource- and time-consuming (12). Patient-derived tumor cell (PDC) models provide unique opportunities for prospective preclinical research. Moreover, we previously demonstrated that PDCs exhibit high degrees of genetic similarity compared to the parental tumors both molecularly and phenotypically (13, 14).

Drug combination therapies can enhance therapeutic efficacy, decrease toxicity, and circumvent both inherent and acquired resistance to standard treatments. Although previous studies revealed dynamic pharmacogenomic interactions across a broad range of tumor types, major gene-drug associations were primarily based on single target agents (13, 15–17). Furthermore, most drug combination suggestions were primarily based on conceptions and algorithms, lacking direct experimental evidences that support such speculations. To this end, we have developed a systematic platform for evaluation of two-drug combinations in 3-dimensionally cultured PDCs (18). While PDC models serve as reliable proxies for examining anti-cancer drug activities, precise estimation of tumor cell populations is also required to predict the patient response within the clinical framework. Therefore, in this study, we performed immunofluorescence-based image analysis to measure and predict the tumor population index in PDC models for two-drug combinational strategies using an HTS system. We found that our systematic platform could identify potential synergistic responses in individual patients with gastric cancer *in situ*.

## Materials and Methods

### Cell Lines and Patient-Derived Cell (PDC) Culture

Human gastric cancer cell lines, AGS, KATOIII, and NCI-N87, were purchased from American Type Culture Collection (ATCC, Manassas, VA, USA), and MKN-45 and SNU-216 were purchased from the Korean Cell Line Bank (Seoul, South Korea). All cell lines were maintained in RPMI 1640 medium supplemented with 1% antibiotic-antimycotic and 10% fetal bovine serum (Gibco, Grand Island, NY, USA). The primary human dermal normal fibroblast adult (HDFa) cell line was purchased from ATCC and maintained in fibroblast basal media supplemented with fibroblast growth kit compounds (ATCC). Surgically removed tumor tissue, biopsy tissue, or malignant ascites were collected from patients with gastric cancer. The protocol was approved by the Institutional Review Board at the Samsung Medical Center. Collected tissue was minced and dissociated enzymatically, and the collected effusions (500–1,000 mL) were divided into 50-mL tubes, centrifuged at 1,700 rpm for 10 min, and washed twice with Dulbecco's phosphate-buffered saline. Cell pellets were added to 75-cm^2^ flasks containing RPMI 1640 medium supplemented with 10% fetal bovine serum, 1% antibiotic-antimycotic (Gibco), 0.5 g/mL hydrocortisone (Sigma Aldrich, St. Louis, MO, USA), 5 μg/mL insulin, and 5 ng epidermal growth factor (Peprotech, Rocky Hill, NJ, USA). The cell lines and PDCs were maintained at 37°C in a humidified atmosphere in a 5% CO_2_ incubator and passaged using TrypLE-Express (Gibco) to detach the cells when they reached 80–90% confluence.

### 3D Cell Culture

Gastric cancer cell lines and PDCs were detached using TrypLE-Express and seeded into 3D culture media consisting of DMEM/F12 supplemented with 10 mM HEPES, 1% antibiotic-antimycotic solution, B27, N2, 1% Glutamax (Gibco), 1 mM *N*-acetyl-L-cysteine (Sigma Aldrich), 10 μg/mL insulin, 20 ng/mL basic fibroblast growth factor, and 50 ng/mL epidermal growth factor (Peprotech). After 4 days, the cells were dissociated into single cells using Accutase (Gibco) and mixed with 0.75% (w/w) alginate for loading onto the micropillar chip.

### Immunofluorescence Staining in Micropillar Chip

The microwell chip composed of polystyrene has 532 complementary microwells and the micropillar composed of poly (styrene-co-maleic anhydride) contains 532 micropillars. The detailed protocol was described in our previous papers (19–21). Fist, 950 nL 3D culture medium was automatically dispensed onto a microwell chip and incubated in a gas-permeable chamber with water in a 37°C incubator. Next, 50-nL spots containing a 1:1 mixture of 70–100 cells and 0.75% alginate were dispensed onto the micropillar chip using ASFA™ Spotter ST (Medical and Bio Device, Suwon, South Korea). After cell dispensing, the micropillar chip was stamped on top of the microwell chip and incubated in the chamber in a 37°C incubator for 3–5 days. The cells cultured under 3D conditions on the micropillar chip were fixed with 4% paraformaldehyde solution (Biosesang, Sungnam, South Korea). After fixation, the micropillar chips were permeabilised with 1% bovine serum albumin in water containing 0.3% Triton-X for 1 h. Each micropillar chip was incubated overnight at 4°C with primary antibodies and secondary antibodies at room temperature for 3 h. The chips were washed in staining buffer and dried in the dark. The following antibodies were used: anti-EpCAM monoclonal antibody (fluorescein isothiocyanate-conjugated, 1:100, Invitrogen, Carlsbad, CA, USA), anti-vimentin (SP20, 1:200, Abcam, Cambridge, UK), anti-HER2 (3B5, 1:100, Invitrogen), anti-MET (3D4, 1:100, Invitrogen), anti-mouse Alexa Fluor 350 (1:300, Thermo Fisher Scientific, Waltham, MA, USA), anti-rabbit Alexa Fluor 594 (1:300, Thermo Fisher Scientific), and Hoechst 33342 (1:1,000, Thermo Fisher Scientific). The stained chips were scanned using an optical scanner device (ASFA™ Scanner ST, Medical and Bio Device) and scanned images were evaluated with image analysis software (S+ analysis, Samsung Electro-Mechanics Co. Ltd., Suwon-si, South Korea).

### HTS Using 532-Micropillar Chip in Gastric Cancer PDCs

The chip layout was designed for screening of 12 compounds in a single micropillar chip, as previously described (19). In the micropillar chip, ~80–100 cells were immobilized with 0.75% alginate. We tested 24 compounds, AZD2281 (olaparib), AZD4547, AZD5363, AZD6094 (volitinib), AZD6244 (selumetinib), AZD1775, everolimus, crizotinib, palbociclib, regorafenib, AZD6738, vemurafenib, cetuximab, herceptin, sunitinib, PF-0299804 (dacomitinib), lapatinib, BEZ235, AZD2014, ribociclib (LEE011), staurosporin (positive control), neratinib, BGJ-398, and pazopanib in two gastric cancer PDCs. A 50-nL droplet of a 1:1 cell mixture of 1.5% alginate and 950-nL droplet of 3D culture media was dispensed with the ASFA™ Spotter ST (MBD). After overnight incubation, a 950-nL droplet of the 24 compounds was also dispensed with the ASFA™ Spotter ST and stamped with the micropillar chip containing the cells. The combined chips were incubated for 5 days at 37°C and 5% CO_2_ in an incubator for the cell viability assay. After incubation, the micropillar chips were stained with staining buffer (MBD-STA50, Medical and Bio Device) containing Calcein-AM (Invitrogen, live cell staining dye) for 1 h in the dark at room temperature. The stained micropillar chips were washed with staining buffer for 30 min and then dried in the dark.

### Combination Drug Screening Using 384-Well Pillar Plates

Combination drug screening using 384-pillar plates was performed by dispensing compounds into a 384-well plate and then sandwiching the 384-pillar plate on the 384-well plate. The drugs were used: herceptin, AZD2281, AZD1775, AZD6738, LEE011, palbociclib, and staurosporin (positive control). The maximum drug concentration was 20 μM (dissolved in dimethyl sulfoxide) and the doses of drug is six with 3-fold dilution ratio. Two-drug-combination effect was estimated by a Combination Index for the Loewe Additivity (22, 23). The CI_50_ is defined by:

$$\begin{array}{l} CI_{50}=\frac{a}{A}+\frac{b}{B} \end{array}$$

where A and B are the IC_50_ values of drugs A and B, respectively. The IC_50_ is the drug concentration at which cell viability is 50% following single drug treatment. a and b are the concentrations of drugs A and B at which cell viability is 50% when A and B are treated in combination.

In practice, a CI_50_ < 1 indicates that the doses of a and b producing a given effect in combination are lower than the expected dose from additive effects and can thus be directly interpreted as synergy. Similarly, a CI_50_ >1 indicates that the doses of a and b producing a given effect in combination are superior to those of the expected doses from additive effects and can thus be directly interpreted as antagonism.

### Western Blotting

Total protein from cell lines and PDCs were lysed in cOmplete Lysis-M buffer solution (Roche, Basel, Switzerland), and protein concentrations were determined using a Quick Start Bradford Protein Assay (Bio-Rad, Hercules, CA, USA). Equal amounts of protein (30 μg) were boiled for 5 min at 90°C and then separated in a 4–12% Bis-Tris gel (Invitrogen) utilizing the Invitrogen Novex gel running apparatus at 110 V for 90 min in MOPS running buffer. Proteins were transferred to a nitrocellulose membrane (Whatman, Maidstone, UK) at 250 mA for 2 h in Transfer buffer (Biosaesang, Seongnam, South Korea) on ice. The membranes were blocked with 5% skim milk in TBS buffer containing 0.1% Tween 20 and incubated overnight at 4°C with specific primary antibodies. The antibodies were anti-HER2 (phospho Tyr1248, 1:1,000, Cell Signaling Technology (CST), Danvers, MA, USA), anti-HER2 (D8F12, 1:1000, CST), anti-cyclin E1 (D7T3U, 1:1,000, CST), anti-cyclin E1 (phosphor Thr62, 1:1,000, CST), and β-actin (C4, 1:3,000, Santa Cruz Biotechnology, Dallas, TX, USA). Horseradish peroxidase-conjugated anti-rabbit or mouse IgG (Bio-Rad) was used as secondary antibody. Signals were detected by chemiluminescence using ECL Western Blotting Substrate (Thermo Fisher Scientific) and visualized using an LAS-4000 (Fujifilm, Tokyo, Japan).

### Targeted Sequencing

Tumors were subjected to target exome sequencing which covers a range of exonic regions of specific genes that are associated with cancer progression. Genomic DNA was shared using a Covaris S220 (Covaris, Woburn, MA) to construct a sequencing library using the SureSelect XT Reagent Kit, HSQ (Agilent Technologies) on target genes. A paired-end sequencing library was purified and amplified with a barcode tag, and the library quality and quantity were determined. Sequencing was carried out using the 100-bp paired-end mode of the TruSeq Rapid PE Cluster kit and TruSeq Rapid SBS kit on HiSeq 2500 sequencing platform (Illumina, San Diego, CA, USA).

### Mutation Calls

The sequenced reads in the FASTQ files were aligned to the human genome assembly (hg19) using the Burrows-Wheeler aligner (BWA). The initial alignment BAM files were subjected to sorting (SAMtools), removing duplicated reads (Picard), locally realigning reads around potential small insertion/deletion and recalibrating base quality score [Genome Analysis Toolkit (GATK)]. We used MuTect to generate high-confidence predictions on mutation calls. Variant Effector Predictor was used to annotate the called mutations.

### Statistical Analysis

Statistical analysis was performed with GraphPad Prism 5.0 software (GraphPad, Inc., San Diego, CA, USA) based on the fluorescence intensity of EpCAM and vimentin, IC_50_ values, and tumor purity. *T*-tests (and non-parametric tests) were used to compare the mean fluorescence intensity of EpCAM and vimentin from gastric cell lines and normal dermal fibroblasts, and to determine actual purity/predicted purity. Statistically significant mean differences between EpCAM/vimentin intensity and tumor purity were indicated as ^***^p < 0.0001.

## Results

### Prediction of Tumor Purity in Gastric Cancer Cell Lines and PDCs

**Figure 1:**
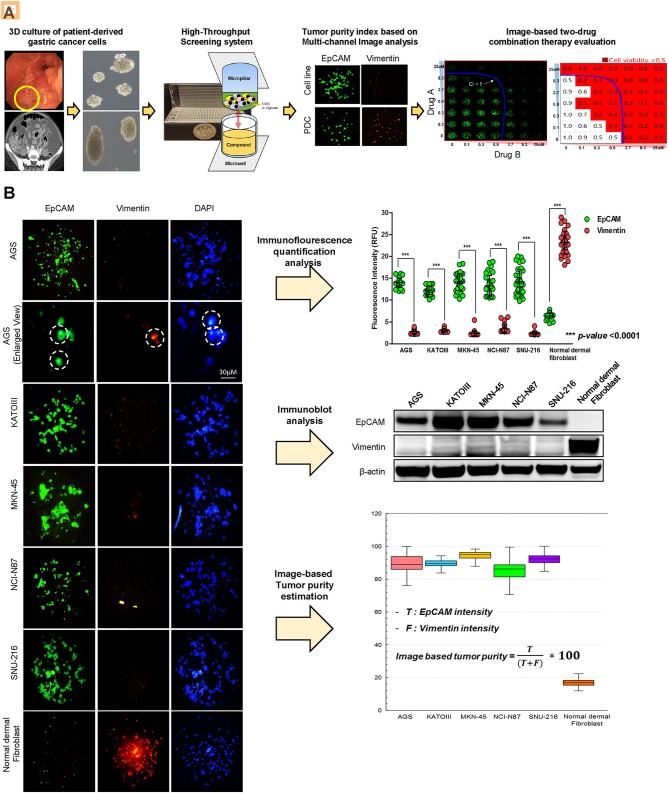
Overview of systematic platform for prediction of tumor purity from patient tumor-derived cells (PDCs) and 3D-based high-throughput drug screening for two-drug combination therapy **(A)** Schematic representation of the generation of patient-derived tumor cell models from tumor tissue or malignant ascites from patients with stage IV gastric cancer. Two-dimensional (2D) cultured monolayer PDCs were seeded with 3D-culture medium. Multi-color antibodies including EpCAM, vimentin, and DAPI were used and fluorescence intensity in various gastric cancer cell lines and PDCs was measured. Tumor purity was predicted. Using PDCs with a proper amount of tumor purity, high-throughput monotherapy, or combinatorial drug screening was performed in a micropillar/microwell chip screening assay. (B) Demonstration of proficient EpCAM expression and deficient vimentin expression in five gastric cancer cell lines (AGS, KATO-III, MKN-45, NCI-N87, SNU-216). DAPI (nuclear blue fluorescent label) was stained to label cell nuclei. EpCAM and vimentin expression levels are depicted as fluorescence intensities (relative fluorescence units, RFU). Demonstration of significantly different expressions of EpCAM and vimentin in five gastric cell lines. Fluorescence intensities of EpCAM and vimentin were measured; EpCAM expression intensity increased when the concentrations of tumor cells proportionately increased.

**Figure 2:**
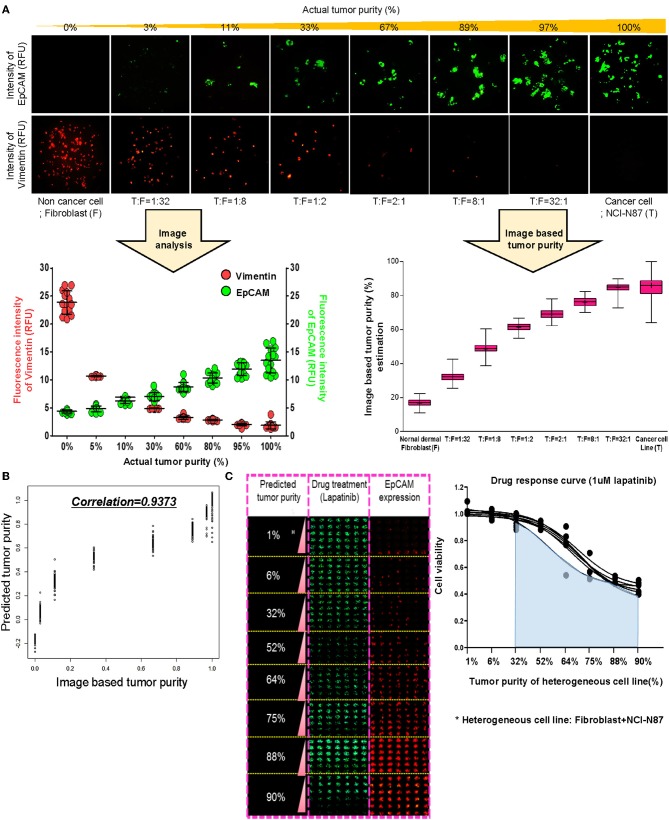
Prediction of tumor purity based on immunofluorescence-based image analysis for gastric PDCs **(A)** Conditions of the various heterogenous cell lines combining NCI-N87 cancer cells with fibroblasts and the fluorescence intensity imaging results. Increased fluorescence intensity of EpCAM and decreased fluorescence intensity of vimentin with increasing concentrations of actual tumor content. Actual tumor purity in mixed cancer cells is calculated as the ratio of EpCAM intensity to combining EpCAM and vimentin intensity. **(B)** From this ratio, the predicted model of tumor purity in PDCs was derived, and the correlation *R*^2^ was 0·9373. **(C)** Drug response curve with lapatinib and various conditions of actual tumor purity in mixed cells.

**Figure 3:**
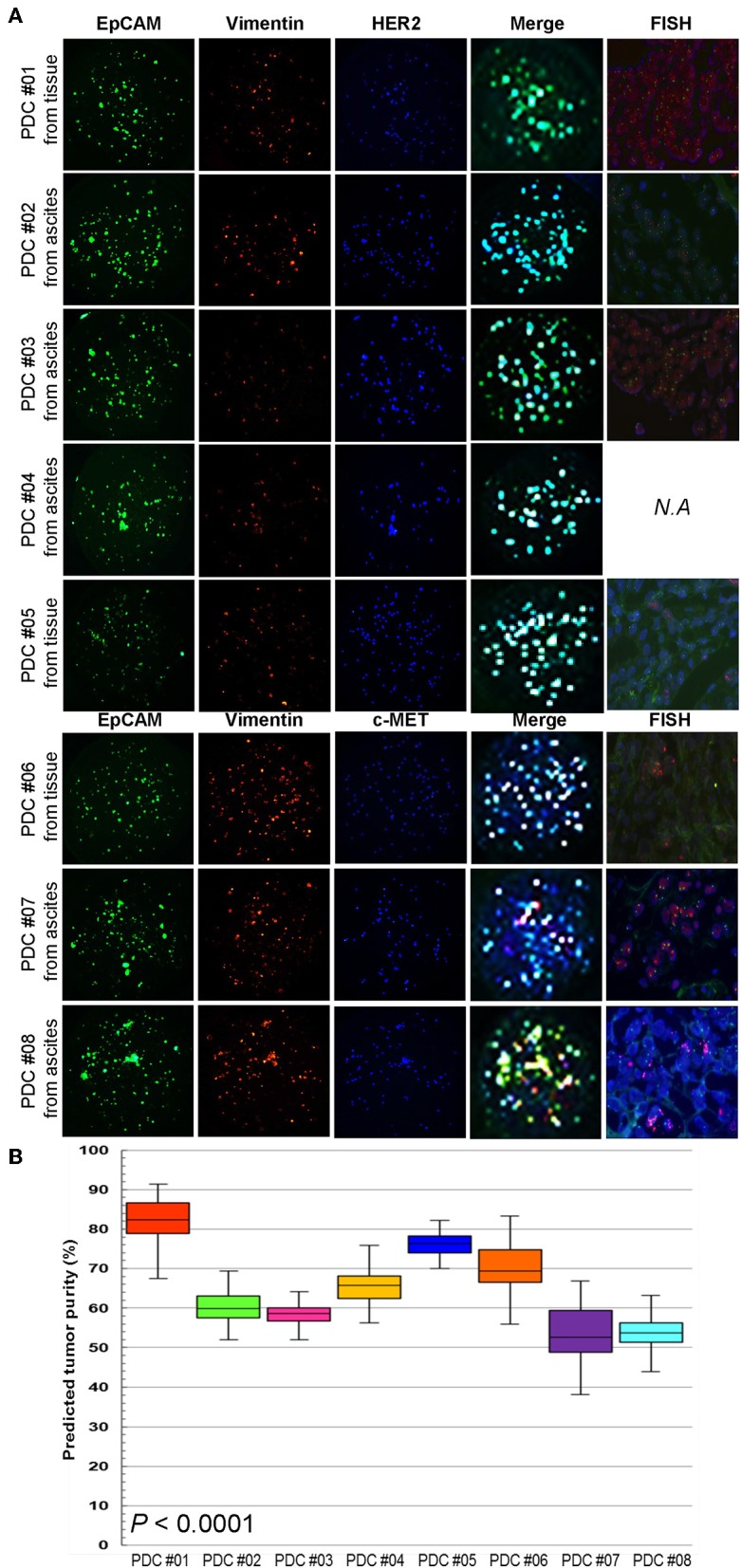
Multi-channel immunofluorescence images of PDCs from 8 gastric cancer patients **(A)** EpCAM, vimentin staining, and merge images for predicting tumor purity in PDCs. The green color shows cancer cell staining and red color is the fibroblast signal. Addition of target oncogene staining to detect HER2 and MET expression in PDCs. The blue color shows HER2 expression in PDC #01-05 and MET expression in PDC #06-08. **(B)** Actual tumor purity and predicted tumor purity.

To establish a systematic HTS platform for evaluating the tumor cell index and two-drug combinational strategy in gastric cancer, we generated a library of PDCs derived from surgically resected gastric tumor specimens or ascites-derived tumor cells (Figure 1A). We have previously demonstrated establishment of 3D cell-based immunostaining protocol. In the present study, the 3D cell-based immunostaining platform has been applied to evaluate gastric cancer purity (19). Multi-color immunofluorescence analyses of EpCAM, vimentin, and DAPI were performed by measuring the fluorescence intensities of respective target molecules in 3D-cultured human gastric cancer cell-lines (AGS, KATOIII, NCI-N87, MKN-45, and SNU-216) on a micropillar chip. Normal dermal fibroblasts were used as a control for detecting non-malignant cells (Figure 1B). Fluorescence intensity analysis showed that all five gastric cancer cell lines were marked by global expression levels of EpCAM, while normal fibroblasts exhibited up-regulation of vimentin expression (Figure 1B). Consistently, immunoblot analysis revealed a significant difference between the protein expression levels of EpCAM and vimentin in both gastric cancer cell lines and fibroblasts. Using the differential intensity levels of EpCAM and vimentin, we formulated an image-based tumor purity estimation to measure the tumor cell index. Notably, when we co-cultured NCI-N87 gastric cancer cells with normal fibroblasts at various cell-to-cell concentrations, we observed a significant correlation between EpCAM and vimentin fluorescent intensity levels (Figure 2A). EpCAM and vimentin expression levels of biological replicates from the mixture of NCI-N87 cancer cells with fibroblasts at different ratios showed significant correlations with minimal variations (Figure 2B). To investigate the minimal requirement for the tumor cellular index to evaluate the appropriate drug response, we seeded a mixture of HER2-positive gastric cancer cells with non-neoplastic cells at various concentrations (from 1 to 90%) and treated the cells with lapatinib. Notably, >30% tumor purity was sufficient for evaluating the therapeutic vulnerability of HER2-positive tumor cells to lapatinib (Figure 2C). To further evaluate the two-drug combinational approach in PDC models, we first determined the tumor cellular index in 5 HER2-positive and 3 MET-positive PDCs (Table 1). Immunofluorescence analysis of EpCAM and vimentin revealed that tumor cells constitute more than 50% of the total cell populations in all 8 gastric PDCs, making them suitable proxies for comprehensive pharmacological analysis (Figures 3A,B).

**Table 1:** Baseline clinical features of patient-derived cancer cells.

**No**.	**Cancer types**	**Date of collection**	**Age**	**Sex**	**Source**	**Pathology**	**Gene**	**Clinical treat**	**CNV gene**
PDC#01	AGC	2016-11-01	61	M	Stomach	Adenocarcinoma	HER2 (3+)	Lapatinib	MYC
PDC#02	AGC	2017-12-15	54	F	Ascites	Tubular adenocarcinoma	HER2 (3+)	Lapatinib	None
PDC#03	AGC	2016-11-01	65	M	Ascites	Adenocarcinoma, moderately differentiated	HER2 (3+)	Lapatinib	ERBB2
PDC#04	AGC	2016-11-25	73	M	Ascites	Tubular adenocarcinoma, moderately differentiated	HER2 (3+)	x	analysis
PDC#05	AGC	2017-12-27	47	M	Lymph node	Tubular adenocarcinoma, moderately differentiated	HER2 (3+), MSH2, MLH1	Herceptin resistant	ERBB2, CCNE1
PDC#06	AGC	2017-10-02	69	F	Stomach	Tubular adenocarcinoma, poorly differentiated	MET (+)	Volitinib	MET, ERBB2
PDC#07	AGC	2017-11-01	63	M	Stomach	Poorly differentiated adenocarcinoma, Favor adenocarcinoma	MET (3+), TSC1, TSC2, MLH1, ATM	Volitinib	MET, MYC
PDC#08	AGC	2017-11-01	43	F	Stomach	Metastatic carcinoma	MET amplification	Volitinib	MET

### Molecular-Guided Two-Drug Combination Treatment in PDC Models

**Figure 4:**
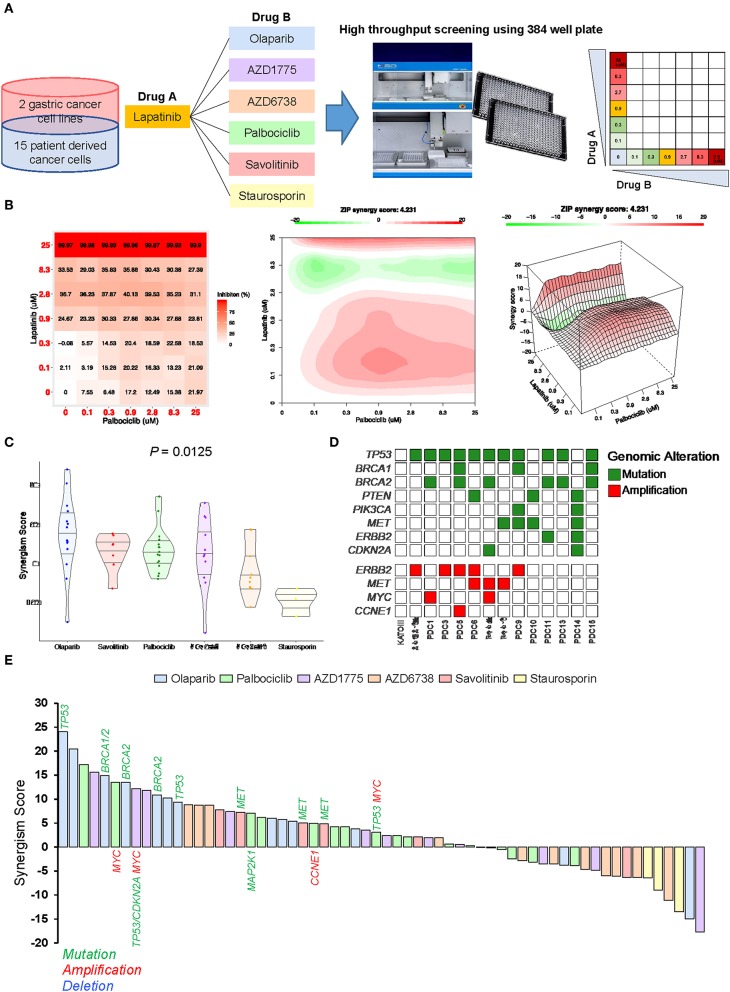
Systematic evaluation of two-drug combination therapy in gastric PDCs **(A)** Representative overview of systematic evaluation of two-drug combination therapy for lapatinib with olaparib, AZD1775, AZD6738, palbociclib, savolitinib, or staurosporin. **(B)** Dose-response matrix for two-drug combination (left panel) and 2D (middle panel) and 3D (right panel) synergy maps. **(C)** Representative violin plots for synergism scores for respective drug combinations. Horizontal lines within the violin plots represent 0.25, 0.50, and 0.75 quantiles. *P* value was calculated by analysis of variance test. **(D)** Genomic landscape of gastric PDCs and gastric cancer cell lines. **(E)** Waterfall plot representation of two-drug combination synergism scores for each drug combination. Genomic mutation, amplification, and deletion are labeled in green, red, and blue colors, respectively.

To identify ideal two-drug combination effects for individual patients, 15 gastric PDCs and 2 cancer cell lines were subjected to lapatinib treatment with olaparib, AZD1775, AZD6738, palbociclib, savolitinib, or staurosporin at various concentrations (Figure 4A). To quantify the degree of two-drug synergistic or antagonistic effects, tumor cellular viabilities were assessed against the expected combination response under non-interaction assumptions using various reference models (24–26). Using the SynergyFinder algorithm, we calculated the synergistic scores for each dose-response matrix among various two-drug combinations (27). Interestingly, although most two-drug combination effects at various concentrations were either additive or synergistic, some combination effects were antagonistic at varying doses (Figure 4B), suggesting that the results of the two-drug combinational approach should be interpreted with caution. Overall, we observed a wide range of drug sensitivities among different drug combinations, demonstrating the highly heterogeneous nature of gastric PDCs (Figure 4C).

As the distinct molecular background across each gastric PDC may explain the dynamic drug profiles, we conducted targeted massively parallel sequencing to identify somatic genomic alterations, including single-nucleotide variations, small insertions/deletions, and copy number variations. Mutations with variant allele frequencies of >5% and >20 reads were considered. Interestingly, most PDCs harbored one or more genomic alterations in key cancer-driver genes, including *TP53, BRCA1, BRCA2, ERBB2, CCNE1*, and *CDKN2A* (Figure 4D). As genomic variations continue to be employed as reliable biomarkers for predicting the clinical response to molecular-guided targeted therapy (28–30), we analyzed pharmacogenomic interactions in gastric PDCs to identify molecular links that dictate the synergistic response to two-drug combination effects. Interestingly, we discovered that tumors with mutations in either *BRCA1* or *BRCA2* were therapeutically susceptible to lapatinib and olaparib combination treatment (Figure 4E). PARP inhibition therapy has shown significant therapeutic success in patients diagnosed with advanced ovarian adenocarcinomas with germline BRCA1/2 mutations. We also found that *MET-*mutant tumors were synergistically sensitive to the combination of savolitinib and lapatinib. Furthermore, somatic mutations in *TP53* and *CDKN2A* with *MYC* genomic amplification showed high sensitivity to the WEE1 inhibitor AZD1775. As p16, p53, and c-Myc are essential regulators of the cell cycle program, deficiency of these key molecules rendered cells more dependent on the Wee1-mediated checkpoint. Consistently, a phase II, single-arm study of AZD1775 monotherapy was conducted (NCT02688907) to evaluate anti-Wee1 treatment in relapsed small cell lung cancer patients with *MYC* family amplification or *CDKN2A* mutation combined with *TP53* mutation. Collectively, our results demonstrate clinical feasibility of molecular-guided two-drug combination therapy.

## Discussion

With recent advancements in sequencing technology, molecular characterization of tumors has been widely conducted to facilitate personalized treatment (2, 31, 32). However, designing effective therapies based on computational analysis alone is confounded by tumor-inherent characteristics, including genomic complexity and intra-tumoral heterogeneity. Molecular and transcriptional heterogeneity of gastric cancer has been characterized using various approaches, led by The Cancer Genome Atlas (TCGA) consortium and multiple global collaborative efforts (2, 3). Furthermore, the HTS system provides additional opportunities for exploring the biological behaviors of cancer cells at the cellular level. Combined, these methods can be used to systematically identify genomic biomarkers and appropriate patient stratifications that can guide development of novel compounds for pre-clinical trials. While these studies have provided comprehensive insights into the dynamic pharmacogenomic interactions across a wide spectrum of cancer types, most gene-drug associations have primarily relied on single agent analysis. Combination therapy has gained considerable attention in the field of oncology in recent years, with numerous studies demonstrating its significant advantage over monotherapies (33–36).

In the present study, we established a systematic method for precisely estimating the tumor cell index to aid in evaluating two-drug combination therapy. Using an immunofluorescence-based approach, we employed numerous gastric cancer cell lines and PDCs to assess tumor cell populations within each given tumor by analyzing the intensity of EpCAM and vimentin expression. It is easy and simple way to estimate the tumor cell population in PDCs by analyzing EpCAM and vimentin immunofluorescence intensity. Furthermore, we could examine the proportion of specific biomarker-expressing tumor cells in each PDC, such as HER-2, EGFR, and MET etc. before anti-cancer drug-sensitivity test.

We discovered that all PDCs harbored >50% tumor cell populations across multiple biological replicates and a minimum cellular index of 30% was required to evaluate reliable drug activities. Moreover, two-drug combination treatments exhibited various drug-drug interactions, varying from synergistic to antagonistic effects. Notably, *BRCA1/2-*mutant tumors were synergistically more susceptible to lapatinib and olaparib combinations, while somatic mutations in *MET* conferred increased sensitivity to savolitinib and olaparib treatment. Interestingly, we showed that tumors harboring genomic alterations in cell cycle-encoding genes, including *MYC, MAP2K1*, and *CCNE1* were synergistically vulnerable to the CDK4/6 inhibitor palbociclib. Finally, MYC-amplified tumors with both TP53 and CDKN2A mutations showed an increased response to AZD1775, suggesting the therapeutic benefits of Wee1-mediated therapy in patients with genomic ablations in the cell cycle program. Because biomarkers including genomic alterations and/or protein expression could be changed as a part of drug-resistant mechanism during and after treatment, promising results from *in vitro* testing do not always translate into *in vivo* efficacy. And limited by small samples of PDCs and available drugs, further drug-combination strategies via optimal biomarker matched needs to be warranted.

We are convinced that our study makes a significant contribution to the literature because we established a systematic method for precisely estimating the tumor cell index to aid in evaluating two-drug combination therapy. Furthermore, our platform provides a real-time relevant tool for personalized treatment through the use of mixed cell populations that are derived from patients without considerable *in vitro* culture. We found that our systematic platform could identify potential synergistic responses in individual patients with gastric cancer *in situ*.

## Conclusions

Collectively, our systematic two-drug HTS platform is integral for addressing current clinical needs to facilitate precision oncology therapy.

## Data Availability Statement

The raw data supporting the conclusions of this manuscript will be made available by the authors, without undue reservation, to any qualified researcher.

## Ethics Statement

This investigation was conducted in accordance with the ethical standards of the Declaration of Helsinki and national and international guidelines and was approved by the Institutional Review Board at Samsung Medical Center in Seoul, Korea.

## Author Contributions

SL, DL, D-HN, and JL conceived and developed study. JS, JK, and BK performed experiments. SL, JS, and JK collected and analyzed data. SL, JS, and JK wrote the manuscript. SK, SP, JP, YP, HL, and WK provided experience in fruitful discussion. JL provided whole project administration and supervision. All the authors discussed the results and commented on the manuscript.

### Conflict of Interest

BK was employed by company MBD Co., Ltd. The remaining authors declare that the research was conducted in the absence of any commercial or financial relationships that could be construed as a potential conflict of interest.
